# Correspondence

**Published:** 1883-03

**Authors:** 


### CORRESPONDENCE.

### "THE VALUE OF GRADUATED PRESSURE IN THE TREATMENT OF DISEASES OF THE VAGINA, UTERUS, OVARIES, AND OTHER APPENDAGES."

TO THE EDITOR OF THE ATLANTA MEDICAL REGISTER :

Dear Sir :—A reprint from your esteemed journal for the present month, with the above title and with the "compliments of the author," Dr. Nathan Bozeman, on the title page, reached me to-day with my regular weekly exchanges. In it I find that Dr. Bozeman defends, by numerous quotations from his published witnesses and by a multitude of statements, his priority to the treatment mentioned in the title. After demolishing, to his apparent satisfaction, the one chief contestant to that honor, Dr. V. H. Taliaferro, of Atlanta, he proceeds to dispose in a like summary manner of me, because, as he asserts, I persisted in ignoring his just claims.

Now, personally, it is a matter of little consequence to me who is entitled to the credit of inventing this *excellent* method, and it would certainly not be worth my while to take any notice of Dr. Bozeman's statements either regarding his priority or my failure to acknowledge it, did he not in the present paper go out of his way to impugn the truth of certain published statements of mine.

So far as the question of priority is concerned, Dr. Bozeman may have *apparent* cause for complaint. Suffice it to say, that I *first* saw a detailed account of the systematic therapeutical tamponade of the vagina for chronic utero-pelvic diseases in a reprint of a paper by Dr. Taliaferro, from the Transactions of the Medical Association of Georgia, for 1878, which was abstracted by me for the *Centralblatt fur Gynekologie* during July 1878, and published in No. 22, October 26, 1878 of that journal. From this article I gathered my first incentive to practice this method, and hence to its author did I give the credit for its introduction. When subsequently informed that Dr. Bozeman claimed the priority, I so stated on several occasions, in print and verbally, but in the absence of proof it was not for me to decide who was right. Dr. Bozeman at a later period offered to show me his peculiar manner of "columning the vagina," and demonstrated it to me to my great satisfaction in several patients in the Woman's Hospital He put the patients in the knee-chest position, used his trivalve speculum and introduced *only dry cotton* balls, which latter seemed to me the chief distinctive point between his and Taliaferro's method of doing substantially the same thing, as it also was a lesson to me who had always used at least the first pledgets soaked in glycerine. But, after all, it was *Taliaferro* who *first* taught me the *principle.*

Judging from Dr. Bozeman's present paper, it seems that a *similar* practice was employed by him in the preparation of vesico-vaginal fistulæ for operation as long ago as 1856. I suppose I may be pardoned if these ancient journal articles were not familiar to me.

But it is not of this question of priority that I wish to speak. The two claimants can fairly be allowed to settle that between them, and I presume Dr. Taliaferro will prove himself able to fight his own battle. I merely wish to state that I had and have no intention of disputing Dr. Bozeman's claim to anything he can *prove.*

But when he descends to questioning the correctness of figures given by me in my article on "Prolapse of the Ovaries" (read before the American Gynæcological Society, September 19th, 1870, and published in Vol. IV. of the Transactions) he deserves correction and rebuke.

He insinuates that, because in the annual report of my service at Mt. Sinai Hospital for 1878, *made to the di-*

*rectors*, I do not speak of prolapse of the ovary as one of the diseases seen by me among the 475 cases under my care during that time, that the 145 cases of that displacement mentioned by me (so he says) as having occurred in 1600 unselected gynæcological cases in the same service must have been seen by me during the nine months between the issuing of my report and the reading of my paper before the Gynæcological Society, which naturally leads "to the conclusion that he (I) must have encountered an unheard of epidemic of prolapsed ovaries at or just before the time he (I) prepared his (my) paper upon the subject." This is no doubt very witty; but the learned gentleman forgets, or appears to forget, that the report in which no mention of prolapsed ovaries was made was prepared solely for *lay-readers* (the Director's and Patrons of the Hospital), that only the *chief* diseases from which the patients suffered were mentioned in the report, and hence prolapse of the ovary, which seldom occurs alone, was not referred to, whereas the chief complaint, such as retroversion, cellulitis etc., was recorded. My case books at the hospital, however, show that several years previously I made note of the condition of the ovaries including prolapse, a special heading having been printed for those organs. Indeed, my attention was called as long ago as 1871 during a private touch course with Dr. Funk in the female venereal ward at the Vienna Hospital to the palpability of the ovaries, normal and pathological, as my notebook of that period still shows. In later reports to the directors of Mt. Sinai Hospital, when my notice had been particularly directed to prolapse of the ovary by my studies in 1879 and I was watching for that affection. I believe I did occasionally give it a prominent place.

But to show the animus of Dr. Bozeman and the looseness of his quotations, I will cite a few lines from my paper on Prolapse of the Ovary, referring to my statistics: "Among 1600 unselected gynæcological cases which have come under my care during the last few years, and of which I have accurate notes, there were 145 *cases in which one or both ovaries were palpable.* In 68 cases the ovaries retained their normal position; *in 77 cases they were pro-*

*lapsed*" But Dr. Bozeman says that I report 145 cases of *prolapse*, and by direct inference gives the reader to understand that all these 1600 cases and 145 cases were seen by me at Mt. Sinai Hospital; whereas I nowhere make such a statement, for quite a number were from my private practice, and some others from a small dispensary to which I was attached before the Mt. Sinai Hospital Outdoor Department was organized. I need but hint at the inference naturally to be drawn from such loose quotations.

Irrespective of the fact that this question of my figures on Prolapse of the Ovary is entirely irrelevent to the subject which Dr. Bozeman has under consideration in his paper, it is as absurd as it is ungentlemanly and unworthy of a man with Dr. Bozeman's claims to fame and standing, to cast reflections on the professional veracity of a colleague, when the same means which enabled him to give a coloring to his insinuations would easily have permitted him to assure himself of their want of foundation.

As for the article by Dr. Bozeman on "Retroversion and Lacerated Cervix" presented to the American Gynæcological Society in September 1878, on which Dr. Bozeman was admitted (I being one of the council) and which appeared in Vol. III of the Transactions, and in which the treatment by "graduated pressure" is fully described, I must plead guilty to never having read it until to day. His charge that I did not give him credit for his "method of columning the vagina with *dry cotton*" in my book (December 1880) so far as I remember, is answered by the fact that at that time, I was not aware through his private demonstration to me at the Woman's Hospital, that *that* was the peculiarity of *his* method. The new edition of my book, now nearly completed, distinctly states the difference between the methods of the two rival claimants. As for the charge that I give credit in my book neither for Dr. B's "designating word 'columning,'" or for Dr. Touszky's term 'tamponade of the vagina,' permit me to say that I do not think the word "column" was invented or patented by Dr. Bozeman, even when a column of cotton in the vagina is intended; and that the term "tamponade of the vagina was known to me years before I had ever heard of Dr. Bozeman or Dr. Touszky. (See Scanzoni, Obstetrics, 1867. Index, under T.)

I had not intended, Mr. Editor, to take up so much of your valuable space. As it is, I have been compelled to pass over many details which would have rendered the actual facts much more clear. But personal discussions of this kind can interest the general profession but little, and only in order that by silence I might not imply consent, have I thought it my duty to intrude upon your readers. When Dr. Bozeman again appears in print I hope that he will omit personalities (which he so much deplores in Dr. Taliaferro's recent article) and confine himself strictly to facts. Groundless insinuations against others, certainly of equal standing with himself in the profession, can but injure his own cause. For my part, I have done with this matter.

Very respectfully,

PAUL F. MUNDE.

20 West 45th Street, New York, January 30th, 1883.

---

TO THE EDITOR OF THE ATLANTA MEDICAL REGISTER:

Dear Sir:—From providential causes wholly unavoidable I have thus far been unable to prepare a reply to the communication of Dr. Nathan Bozeman in December and January numbers of the REGISTER, but will do so at an early day.

In the mean time I beg that your readers will bear in mind and file away the number containing Dr. Bozeman's paper for reference when they have my reply at hand. Very truly yours,

V. H. TALIAFERRO.

Atlanta, February 21, 1883.

